# A Rare Case of Effusive Constrictive Cholesterol Pericarditis: A Case Report and Review

**DOI:** 10.1155/2013/439505

**Published:** 2013-04-01

**Authors:** Van W. Adamson, Jennifer N. Slim, Kenneth M. Leclerc, Ahmad M. Slim

**Affiliations:** ^1^Cardiology Service, San Antonio Military Medical Center, Fort Sam Houston, San Antonio, TX 78234, USA; ^2^Internal Medicine Service, San Antonio Military Medical Center, Fort Sam Houston, San Antonio, TX 78234, USA

## Abstract

Effusive constrictive cholesterol pericarditis is exceedingly rare. Most cases have an unclear etiology but can be associated with rheumatoid arthritis, tuberculosis infection, and hypothyroidism. The hallmark of the effusion is the distinctively high levels of cholesterol. We present the case of a 68-year-old male with prolonged symptoms of dyspnea with associated moderate pericardial effusion that were later determined to be constrictive effusive etiology, and the patient was referred for stripping with pathologic cholesterol crystal formation on pathology review.

## 1. Case Presentation

**Figure 1:**
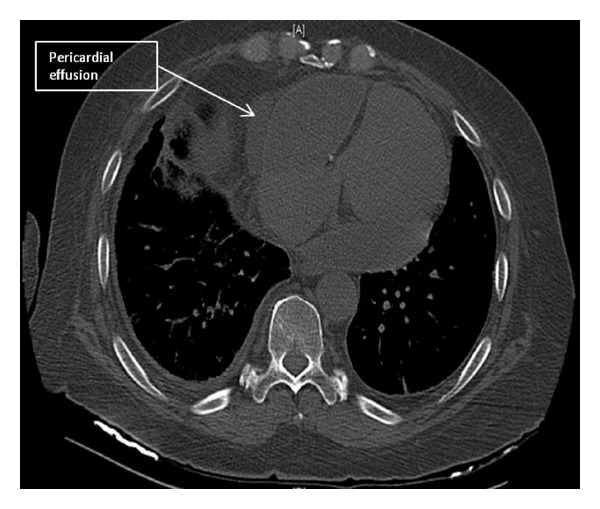
Axial noncontrast CT image of the thorax showing the heart with pericardial effusion.

This is a case of a 68-year-old male with a history significant for rheumatoid arthritis and a moderate pericardial effusion discovered incidentally on a CT scan of his chest during the initial workup for worsening dyspnea (Figure 1). Over the next 18 months, he developed increasing lower extremity edema and dyspnea with activity. Symptoms at that time showed modest response to oral diuretics. Serial echocardiograms, during the 18-month evaluation period, were performed periodically to evaluate his worsening dyspnea and generalized edema. Despite his worsening symptoms, the echocardiograms showed no decrement in the systolic or diastolic function with minimal increase in size of the effusion with no end-diastolic chamber collapse, interventricular dependence, or spectral Doppler variation suggestive of tamponade physiology. Physical exam revealed a normotensive male that was not tachycardic. There was a pericardial knock and distended jugular veins. He had coarse breath sounds bilaterally and mild abdominal distention with hepatomegaly. Extremity exam showed deep pitting edema to his thighs and small joint deformities associated with his known rheumatoid arthritis. A directed echocardiogram for evaluation of constrictive pericarditis showed a hyperdynamic left ventricle. Right ventricular collapse was evident along with respirophasic ventricular septal motion. These findings along with bilateral atrial enlargement, in the absence of restrictive pattern on diastolic function evaluation, were suggestive of possible pericardial constriction rather than tamponade.

**Figure 2:**
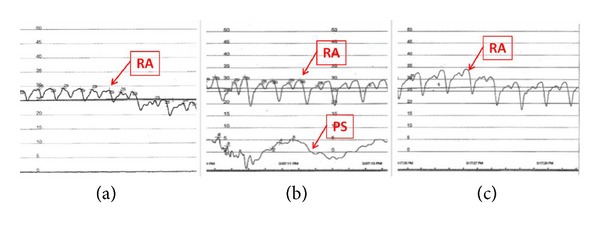
Hemodynamics noted during the right heart catheterization. (a) Elevated right atrial (RA) pressure showing respiratory variations. (b) Elevated RA pressure despite decreased pericardial space (PS) pressure immediately after evacuation of pericardial fluid. (c) Elevated RA pressure despite full evacuation of fluid.

**Figure 3:**
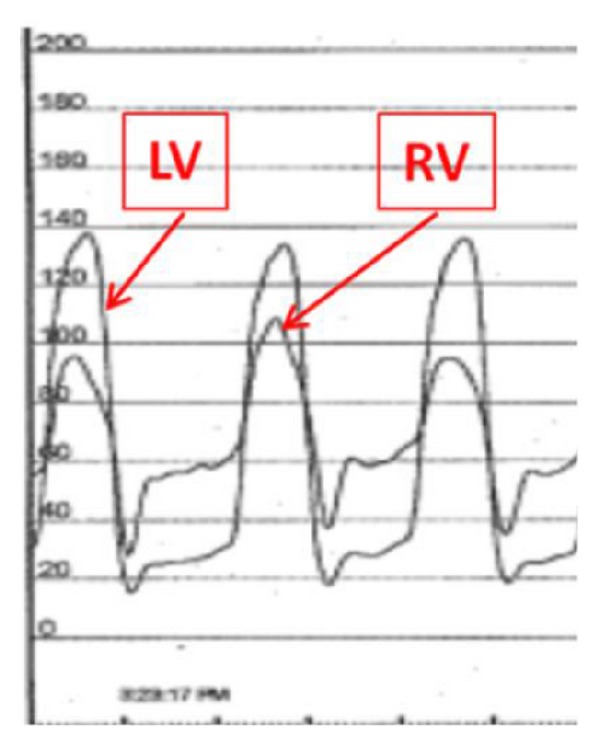
Hemodynamics during right heart catheterization showing persistence ventricular interdependence between the left ventricle (LV) and the right ventricle (RV) after evacuation of pericardial fluid.

The patient continued to worsen over the next two years. He underwent a diagnostic pericardiocentesis with cardiac and pericardial pressure monitoring. Pericardial pressures were elevated at 30 mmHg prior to drainage of fluid. There was hemodynamic evidence of equalization of diastolic pressures in all chambers. There were a positive Kussmaul sign and significant ventricular interdependence. After pericardiocentesis, pressures in the pericardial space decreased to less than 5 mmHg. Despite this extracardiac change in pressure, there were no changes in the intracardiac pressures (Figure 2). There continued to be a prominent square root sign on both right and left ventricular tracings. Right atrial pressure tracings now demonstrated a blunted X descent and rapid Y descent (Figure 3). These findings supported a diagnosis of constrictive pericarditis as the etiology of heart failure in this patient. Pericardial fluid was bloody and revealed no infectious etiology on analysis and cultures including acid fast bacillus, with total protein content of 5.1 g/dL, glucose of 4 mg/dL, LDH 5640 u/L, and a total cholesterol of 211 mg/dL. In view of the patient's significant symptomatology, he was referred for pericardial stripping in a high volume surgical center. Pathology report of the pericardial tissue, obtained from the surgical center where the stripping took place, revealed* degenerating blood with focal areas of cholesterol crystal formation.*

## 2. Discussion

Effusive constrictive cholesterol pericarditis is exceedingly rare. Most cases have an unclear etiology but can be associated with rheumatoid arthritis, tuberculosis infection, and hypothyroidism [1]. The hallmark of the effusion is the distinctively high levels of cholesterol. Effusions are large and can cause compression and adherence of the visceral pericardium to the heart [1]. The classic findings in effusive-constrictive pericarditis are persistently elevated right atrial pressures after intrapericardial pressures have been reduced to normal levels after removal of intrapericardial fluid [2]. The clinical course after drainage is one of reaccumulation of pericardial fluid over time. Patients normally present with signs and symptoms of acute heart failure. In a few cases after pericardiocentesis, constrictive pericarditis resolves spontaneously over time. Many patients undergo pericardial stripping of the visceral pericardium which has a surgical morbidity and mortality as high as 21% [3]. The medical literature is sparse about nonsurgical treatment [2]. This case illustrates a presentation of effusive constrictive cholesterol pericarditis. It was initially detected on chest CT with pathophysiology demonstrated by echocardiogram and invasive hemodynamics. Final pathology was confirmed by pericardial biopsy.

## 3. Conclusion

Effusive constrictive cholesterol pericarditis is a rare form of this pericardial syndrome. This condition has a relapsing course that in most cases requires pericardiectomy for definitive treatment. This syndrome should be considered in individuals with chronic inflammatory conditions with recurrent pericarditis and pericardial effusions.
